# Age-Related Responses in Circulating Markers of Redox Status in Healthy Adolescents and Adults during the Course of a Training Macrocycle

**DOI:** 10.1155/2015/283921

**Published:** 2015-04-06

**Authors:** Athanasios Zalavras, Ioannis G. Fatouros, Chariklia K. Deli, Dimitris Draganidis, Anastasios A. Theodorou, Dimitrios Soulas, Yiannis Koutsioras, Yiannis Koutedakis, Athanasios Z. Jamurtas

**Affiliations:** ^1^Department of Physical Education & Sport Science, University of Thessaly, Karies, 42100 Trikala, Greece; ^2^Department of Physical Education & Sport Science, University of Thrace, 69100 Komotini, Greece; ^3^Department of Kinesiology, Institute for Research & Technology, Thessaly, 42100 Trikala, Greece; ^4^School of Sport, Performing Arts & Leisure, Wolverhampton University, Gorway Road, Walsall, West Midlands WS1 3BD, UK

## Abstract

Redox status changes during an annual training cycle in young and adult track and field athletes and possible differences between the two age groups were assessed. Forty-six individuals (24 children and 22 adults) were assigned to four groups: trained adolescents, (TAD, *N* = 13), untrained adolescents (UAD, *N* = 11), trained adults (TA, *N* = 12), and untrained adults (UA, *N* = 10). Aerobic capacity and redox status related variables [total antioxidant capacity (TAC), glutathione (GSH), catalase activity, TBARS, protein carbonyls (PC), uric acid, and bilirubin] were assessed at rest and in response to a time-trial bout before training, at mid- and posttraining. TAC, catalase activity, TBARS, PC, uric acid, and bilirubin increased and GSH declined in all groups in response to acute exercise independent of training status and age. Training improved aerobic capacity, TAC, and GSH at rest and in response to exercise. Age affected basal and exercise-induced responses since adults demonstrated a greater TAC and GSH levels at rest and a greater rise of TBARS, protein carbonyls, and TAC and decline of GSH in response to exercise. Catalase activity, uric acid, and bilirubin responses were comparable among groups. These results suggest that acute exercise, age, and training modulate the antioxidant reserves of the body.

## 1. Introduction

Strenuous exercise increases oxygen consumption in the working muscles by 100- to 200-fold above resting levels resulting in a marked rise of reactive oxygen and nitrogen species (RONS) formation [1]. Several molecular mechanisms contribute to RONS release during exercise [2] which in turn activate enzymatic and nonenzymatic antioxidants against RONS [3]. When RONS production overrides antioxidant defence oxidative stress develops in a tissue-specific manner, thereby altering cellular redox status [3]. Redox status perturbations appear to be essential for regulation of redox-sensitive intracellular signalling pathways, mediating exercise-induced inflammatory responses and skeletal muscle regeneration [4].

Puberty is a period during which rapid growth, development of sexual characteristics, and reproductive competence occur [5] due to upregulation of anabolic and other hormones [6, 7]. It is uncertain whether puberty interferes with antioxidant activity of adolescents. A reduction of antioxidant activity observed in response to growth hormone deficiency in adolescents was reversed following growth hormone replacement treatment [8]. Moreover, testosterone was able to inhibit* in vitro* neutrophil prooxidative capability [9] and in Japanese adolescents was negatively correlated with oxidative stress indices [10]. These findings and data from other investigations suggest that puberty tends to upregulate antioxidant system [11].

Intense exercise induces an inflammatory response and is associated with increased oxidative stress and antioxidant activity [4, 12]. Previous research has reported both increases and reductions of oxidative stress markers and antioxidant status indices in children and adolescents in response to intense exercise [13–16]. Differences in exercise mode may partly explain these discrepancies (weight-bearing activities versus swimming). Although adolescents develop an inflammatory response to exercise like adults [16, 17] that leads to increased oxidative stress markers and antioxidant activity, one should be very cautious in extrapolating data derived from adult literature to children or adolescents. Younger individuals exhibit more rapid VO_2_ kinetics, which is in accordance with the concept that children and adolescents are more aerobic, with a greater mitochondrial capacity for oxidative phosphorylation and less anaerobic than adults during exercise [18, 19]. These characteristics in children increase the likelihood to generate more RONS in response to exercise than adults [20]. If this hypothesis is correct, then exercise should also elicit a marked antioxidant response. Available data on the effects of acute exercise on antioxidant activity of adolescents is scarce. Intense prolonged running left total antioxidant capacity reduced glutathione and antioxidant enzymes unaffected [14], whereas vigorous swimming upregulated antioxidant activity in adolescents [16].

RONS regulate exercise-induced physiological adaptations such as mitochondrial biogenesis, management of cellular stress by mediating several intracellular signaling pathways (e.g., NF-*κ*B pathway, cytokine expressions pathways) [4, 21]. Although we know that exercise training upregulates antioxidant potential in adults and aged individuals [22, 23], very limited information exists for adolescents and children. According to the theory of hormesis acute increases of RONS in response to single exercise sessions precondition skeletal muscle and other tissues by adapting their redox status to these multiple oxidative challenges and chronically elevate their antioxidant defense mechanisms [24]. However, this theory has not been documented in adolescents yet, since the few studies conducted so far have produced contradictory results. Prolonged intense training with long-distance running in adolescents did not increase basal antioxidant activity [14], whereas swimming training did [25].

Thus, the purpose of this investigation was (i) to determine whether a running training macrocycle affects basal oxidative stress development and antioxidant status during early puberty and (ii) to examine if adults and adolescents demonstrate similar or different redox status adaptations to equal volumes of running training. Results from the present study indicate that acute exercise, age, and training modulate the antioxidant reserves of the body.

## 2. Materials and Methods

### 2.1. Participants

Thirty adults and 30 adolescents volunteered to participate in the study. Selection criteria included (a) the fact that control subjects (adults and adolescents) should not be involved in organized sports, (b) absence of musculoskeletal and/or other health problems, (c) no use of nutritional supplements and medications before (≥6 months) and during the study, (d) the fact that participants were nonsmokers, and (e) the fact that adolescents were at Tanner stages 2-3 of pubertal development throughout the study. Eight adults and adolescents were excluded from the study due to failure to comply with the inclusion criteria. Accordingly, 22 adults (19 men and 3 women) and 24 adolescents (19 boys and 5 girls) completed the study. The anthropometric, physiological, and training characteristics of participants are shown in Table 1. Adult participants and adolescent participants' parents/guardians signed a voluntary consent. The procedures were conformed to the Helsinki declaration of 1975 and were approved by the Human Subjects Committee of the local University (Ref#:66/10-4-2008).

### 2.2. Experimental Design

In order to determine redox status changes of adolescent long-distance runners during an 11-month (September through July), two-peaked, training macrocycle and compare them to corresponding changes of adult long-distance runners following the same training regimen, a four-group, repeated measures design was employed. Two control groups were used to control seasonal variations of dependent variables. Participants were assigned to one of the following groups: (a) control adolescents (UAD, *N* = 11, did not participate in training), (b) trained adolescents (TAD, *N* = 13, participated in training), (c) control adults (UA, *N* = 10, did not participate in training), and (d) trained adults (TA, *N* = 12, participated in training). UAD and UA abstained from any structured training program (except for activities performed at school during Physical Education classes for UAD) and were involved only in recreational sports. During their initial visit to the laboratory (end of August), participants had their anthropometric profile (body mass, height, and body composition), stage of sexual maturity (adolescents only), and maximal oxygen consumption (VO_2max_) measured. In a second visit (5 days later), participants in all groups performed a time-trial test to exhaustion while blood was collected at pre- and posttest as well as at one hour (h) of recovery. These measurements were repeated following the conclusion of each competitive period (end of February and mid-July) in exactly the same order. All measurements and blood sampling at mid- and end-season were performed seven days after the last training session.

### 2.3. Training

**Figure 1:**
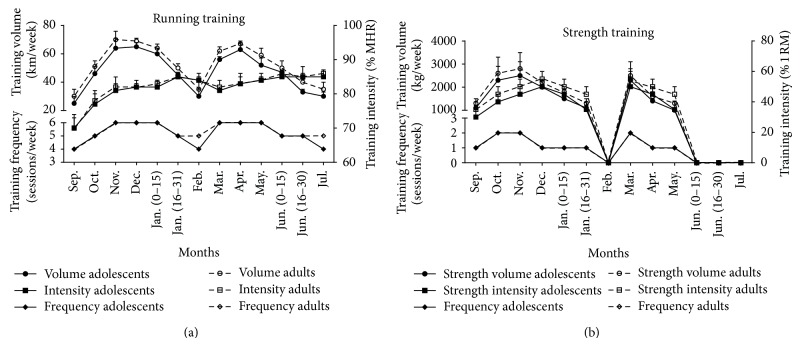
Average weekly volume, intensity, and frequency of running and strength training during the experimental period for the adolescent and adult training groups. MHR, maximal heart rate; 1-RM, one maximal repetition. Data are presented as mean ± SD.

Training followed the annual periodization plan for long-distance runners and followed an individualized program that consisted of two competitive periods. Participants started following a 6-week off-season period (mid-July–end of August) consisting of minimal training. Adults and adolescent athletes followed similar programs consisting mainly of continuous (65–85% VO_2max_) or interval aerobic running. Training regimens were supplemented periodically with speed training (one session/week) and strength training with resistance exercises once or twice a week. The macrocycle consisted of a first preparatory phase (September to mid-January), a first tapering phase (the second part of January), a first competitive phase (February) that included 5 races for the TA group and 4 races for the TAD group, a second preparatory phase (March to May), a second tapering phase (first half of June), and a second competitive phase (second part of June and July) that included 8 races for the TA group and 6 races for the TAD group. Average weekly training volume, intensity, and frequency of the training macrocycle are shown in Figure 1. Running intensity was monitored by telemetry (Polar, Electro Oy, Finland).

### 2.4. Exercise Testing

During exercise testing, participants ran for 45 min on a treadmill at intensity equal to ~75% of their VO_2max_. Thereafter, intensity (speed) increased to 90% VO_2max_ and participants ran until volitional fatigue. This test has been shown to induce a marked elevation of oxidative stress [26]. Participants had access to water ad libitum during testing. Heart rate, VO_2_, and ratings of exertion on a Borg scale (6–20) were monitored throughout testing.

### 2.5. Measurements

All measurements took place in the morning (08.00–10.00 a.m.). Female participants were tested during the early follicular phase of their menstrual cycle (days 2–8) to minimize variance in estrogen levels [27]. Body mass was measured to the nearest 0.5 kg with subjects lightly dressed and barefoot (Beam Balance 710, Seca, UK, Serial #: 940298) and standing height was measured to the nearest 0.5 cm (Stadiometer 208, Seca, UK, Serial #: 940298). Percentage body fat was calculated from 7 skinfold measures (average of 2 measurements at each site), using a Harpenden caliper (John Bull, St. Albans, UK). Maximal oxygen consumption (VO_2max_) was determined using a treadmill test to exhaustion. The protocol began at 7–10 km·h^−1^ (0% slope) and progressed by 0.5 km and increased each minute until VO_2max_ was reached. Criteria used to determine VO_2max_ were (i) subjects' exhaustion, (ii) a <2 mL·kg^−1^·min^−1^ increase in VO_2_ with an increase in work rate, (iii) a respiratory exchange ratio greater than or equal to 1.10, and (iv) a heart rate within 10 bpm of the theoretical maximum heart rate (220 - age). Respiratory gas variables were measured using a metabolic cart (Vmax29, Sensormedics, USA, Serial #: 00363), which was calibrated before each test using standard gases of known concentration. Exercise heart rate was monitored by telemetry (Polar Tester S610TM, Electro Oy, Finland, Serial #: 190030907). Finally, the velocity at maximal oxygen consumption (vVO_2max_) and the velocity at anaerobic threshold (vTh) were recorded. The vTh was determined by the V-slope method [28] while vVO_2max_ was defined according to Billat et al. [29].

### 2.6. Dietary Analysis

To establish that macronutrient and antioxidant intake levels were similar in all groups, participants were asked to record their diet for five consecutive days once a month. Participants were provided with a written set of guidelines for monitoring dietary consumption and a record sheet for recording food intake. Diet recalls were analyzed with ScienceFit Diet 200A (Science Technologies, Athens, Greece).

### 2.7. Blood Sampling and Assays

Participants were asked not to engage in any intense physical activity and not to consume alcohol or caffeine products for at least 72 h before reporting to the laboratory. For plasma collection, a portion of blood was placed in separate tubes mixed with EDTA (20 *μ*L/mL of blood) and centrifuged (1370 g, 10 min) and the supernatant was transferred into Eppendorf tubes that were stored at −80°C for later measurement of protein carbonyls (PC), thiobarbituric acid reactive substances (TBARS), and total antioxidant capacity (TAC). Packed erythrocytes were diluted with distilled water (1 : 1 v/v), vortexed vigorously, and centrifuged (4000 g, 15 min) for red blood cell lysate preparation and the resultant supernatant was transferred into Eppendorf tubes that were stored at −80°C for later analysis of GSH concentration and catalase activity. Finally, another portion of blood was collected in plain tubes, left at room temperature for 20 min to clot, and centrifuged (1370 g, 10 min) for serum separation and the supernatant was transferred into Eppendorf tubes that were stored at −80°C for later determination of bilirubin and uric acid.

TBARS were measured according to Keles et al. [30]. Protein carbonyls were analyzed according to procedures described by Patsoukis et al. [31]. Total antioxidant capacity (TAC) was measured as described by Janaszewska and Bartosz [32]. For GSH measurement [33], erythrocyte lysates (20 *μ*L) were treated with 5% TCA mixed with sodium potassium phosphate (660 *μ*L, 67 mM, pH 8.0) and 5,5′-dithiobis-2 nitrobenzoate (330 *μ*L, 1 mM). Samples were then incubated (in the dark, room temperature, 45 min) and their absorbance was measured at 412 nm. Catalase activity was measured as described by Aebi [34]. Hb was determined with a commercially available kit (Dutch Diagnostics BV, Netherlands). Bilirubin and uric acid were measured in a Clinical Chemistry Analyzer Z 1145 (Zafiropoulos Diagnostica, Athens, Greece, Serial #: 52079) with commercially available kits (Zafiropoulos, Athens, Greece). Each variable was analyzed in duplicate on the same day. Inter- and intra-assay coefficients of variation for all assays ranged from 2.4% to 6.8% and from 2.8% to 7.1%, respectively. Spectrophotometric assays were performed on a Hitachi 2001 UV/VIS (Hitachi Instruments Inc., USA, Serial #: 1904-022) in triplicate.

### 2.8. Statistical Analysis

Data normality was examined using the Shapiro-Wilk test and was not found to differ significantly from normal. A repeated measures (on time) ANOVA (on age and training status) and a Tukey post hoc test were used to analyze the data. SPSS was used for all analyses (SPSS Inc., Chicago, Ill) The level of statistical significance was set at *P* < 0.05.

## 3. Results 

**Figure 2:**
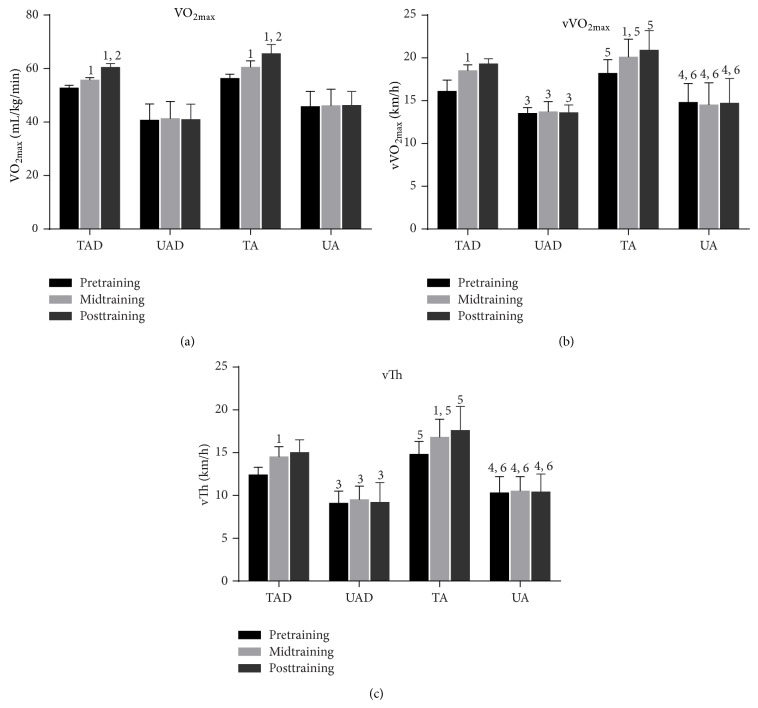
Performance changes during the experimental period. VO_2max_, maximal oxygen consumption; vVO_2max_, velocity at maximal oxygen consumption; vTh, ventilatory threshold; TAD, trained adolescents; TA, trained adults; UAD, untrained adolescents; UA, untrained adults; ^1^a significant difference with baseline (*P* < 0.05); ^2^a significant difference between midseason and end-season (*P* < 0.05); ^3^a significant difference between adolescent groups at the corresponding time point (*P* < 0.05); ^4^a significant difference between adult groups at the corresponding time point (*P* < 0.05); ^5^a significant difference between trained groups at the corresponding time point (*P* < 0.05); ^6^a statistical difference between control groups at the corresponding time point (*P* < 0.05).

TAD and TA had lower body mass, height, BMI, and %fat compared to UAD and UA, respectively (Table 1). The two training groups exhibited a higher total daily energy intake than their age-matched untrained groups but no differences were detected among groups in regard to their antioxidant nutrients intake (Table 2). TAD and TA had a greater VO_2max_ (*P* < 0.05), vVO_2max_ (*P* < 0.05), and vTh (*P* < 0.05) than UAD and UA (Figure 2). VO_2max_, vVO_2max_, and vTh increased (*P* < 0.05) both in TAD and TA at midseason and at the end of the training season compared to baseline values but no differences were detected between mid- and postseason in both groups for vVO_2max_ and vTh (Figure 2). All fitness parameters remained unchanged in the two control groups (Figure 2).

### 3.1. Redox Status Responses

#### 3.1.1. Acute Exercise Responses

**Figure 3:**
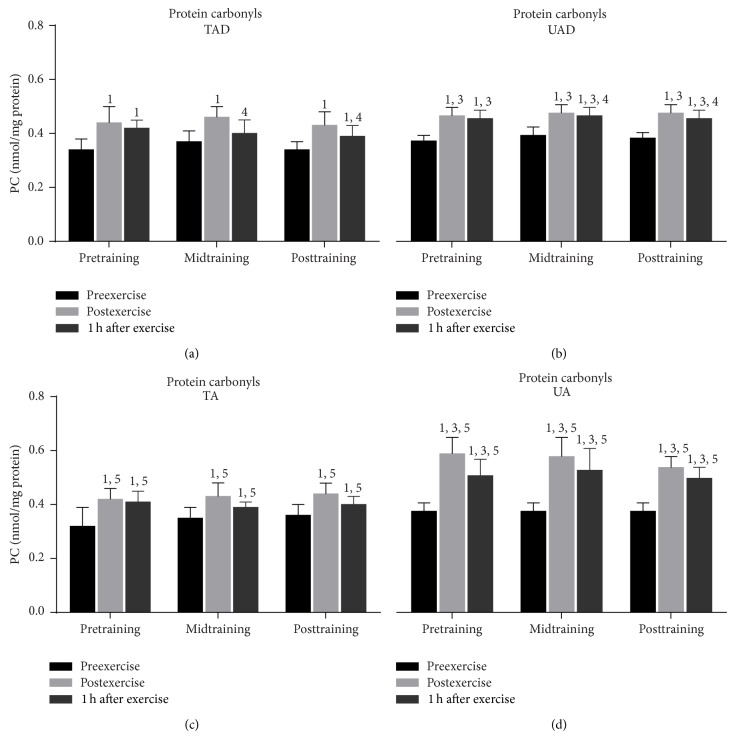
Changes of protein carbonyls in response to acute exercise and training in adolescents and adult participants. PC, protein carbonyls; h, hour; TAD, trained adolescents; TA, trained adults; UAD, untrained adolescents; UA, untrained adults; ^1^significant (*P* < 0.05) difference with baseline values at rest; ^3^significant (*P* < 0.05) difference between untrained adolescents and untrained adults at the corresponding time point; ^4^significant (*P* < 0.05) difference between trained and untrained adolescents at the corresponding time point; ^5^significant (*P* < 0.05) difference between trained and untrained adults at the corresponding time point.

**Figure 4:**
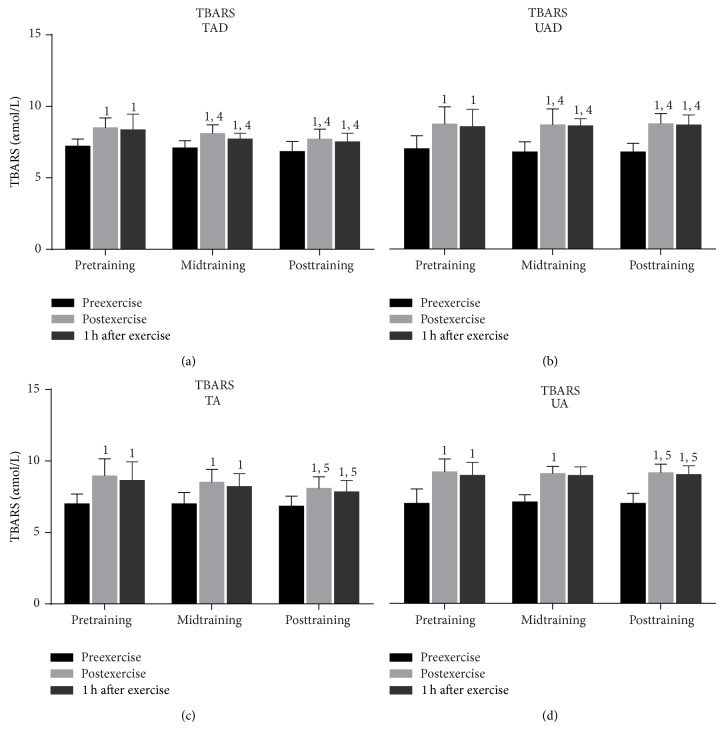
Changes of TBARS in response to acute exercise and training in adolescents and adult participants. TBARS, thiobarbituric acid reactive substances; h, hour; TAD, trained adolescents; TA, trained adults; UAD, untrained adolescents; UA, untrained adults; ^1^significant (*P* < 0.05) difference with baseline values at rest; ^4^significant (*P* < 0.05) difference between trained and untrained adolescents at the corresponding time point; ^5^significant (*P* < 0.05) difference between trained and untrained adults at the corresponding time point.

Exercise increased (*P* < 0.001) PC concentration (at pretraining, midtraining, and posttraining; Figure 3) and TBARS (at pretraining, midtraining, and posttraining; Figure 4) in all groups immediately after exercise. Both TBARS and PC remained significantly above resting values 1 h postexercise in all groups (except for TAD at midtraining; Figure 3).

**Figure 5:**
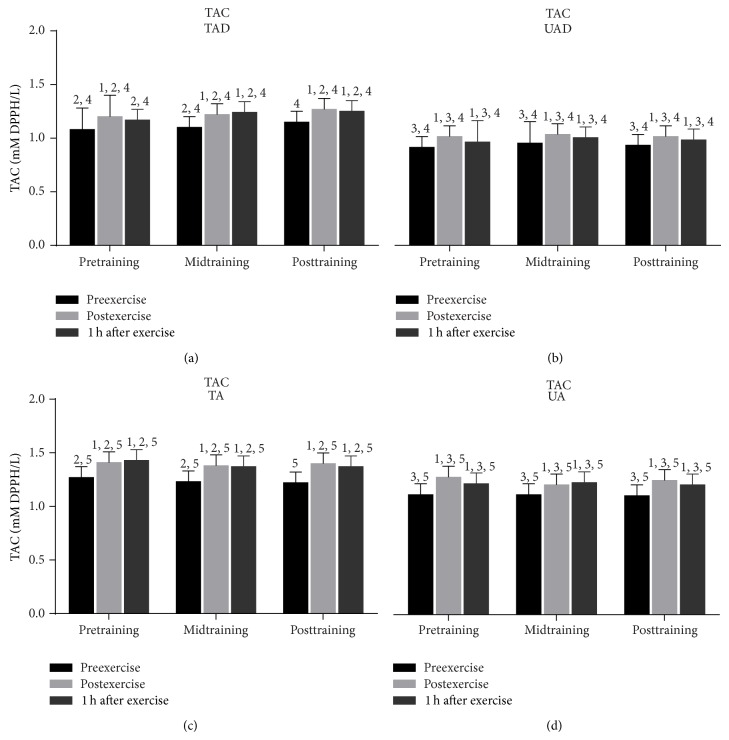
Changes of total antioxidant capacity in response to acute exercise and training in adolescents and adult participants. TAC, total antioxidant capacity; h, hour; TAD, trained adolescents; TA, trained adults; UAD, untrained adolescents; UA, untrained adults; ^1^significant (*P* < 0.05) difference with baseline values at rest; ^2^significant (*P* < 0.05) difference between trained adolescents and trained adults at the corresponding time point; ^3^significant (*P* < 0.05) difference between untrained adolescents and untrained adults at the corresponding time point; ^4^significant (*P* < 0.05) difference between trained and untrained adolescents at the corresponding time point; ^5^significant (*P* < 0.05) difference between trained and untrained adults at the corresponding time point.

Acute exercise induced a marked rise (from 8.2% to 14.5%) of TAC in all groups immediately after exercise throughout the experimental period (at pretraining, midtraining, and posttraining; Figure 5). TAC remained elevated 1 h after exercise (from 5.0% to 13.0%) in all groups throughout the experimental period except for TAD at pretraining.

**Figure 6:**
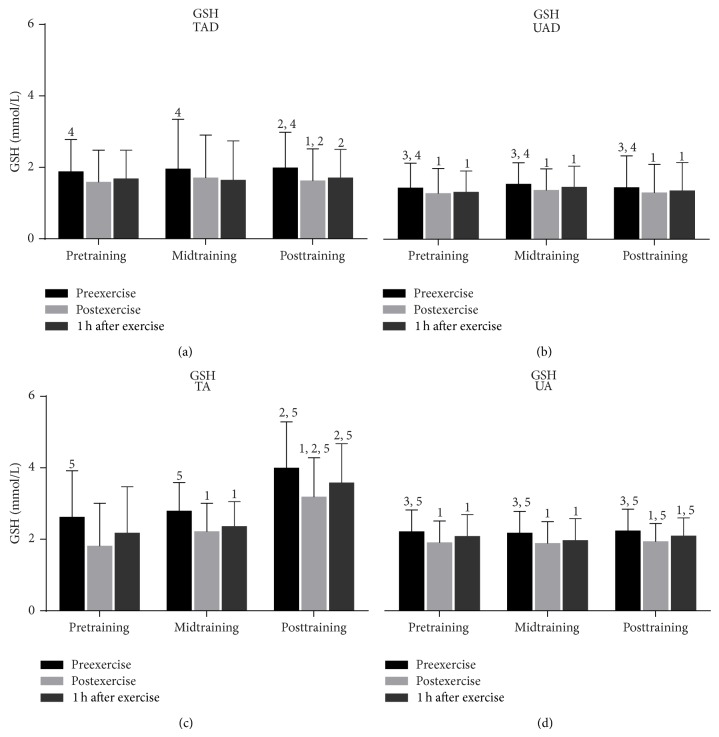
Changes of reduced glutathione in response to acute exercise and training in adolescents and adult participants. GSH, reduced glutathione; h, hour; TAD, trained adolescents; TA, trained adults; UAD, untrained adolescents; UA, untrained adults; ^1^significant (*P* < 0.05) difference with baseline values at rest; ^2^significant (*P* < 0.05) difference between trained adolescents and trained adults at the corresponding time point; ^3^significant (*P* < 0.05) difference between untrained adolescents and untrained adults at the corresponding time point; ^4^significant (*P* < 0.05) difference between trained and untrained adolescents at the corresponding time point; ^5^significant (*P* < 0.05) difference between trained and untrained adults at the corresponding time point.

Although GSH (Figure 6) remained declined in TAD in response to exercise only after exercise at posttraining (*P* = 0.003), it declined in UAD immediately after exercise (10.4% at pretraining: *P* = 0.000; 11% at midtraining, *P* = 0.000; 9.6% at posttraining, *P* = 0.000) and remained below resting values 1 h after exercise at all times (8.3% at pretraining: *P* = 0.011; 6% at midtraining, *P* = 0.001; 6% at posttraining, *P* = 0.000). In UA, exercise reduced GSH immediately (14% at pretraining, *P* = 0.000; 13% at midtraining, *P* = 0.000; 13.2% at posttraining, *P* = 0.000) and 1 h after exercise (6% at pretraining, *P* = 0.000; 9.4% at midtraining, *P* = 0.000; 6.4% at posttraining, *P* = 0.002) throughout the experimental period. However, in TA, GSH remained unaltered in response to exercise at pretraining and declined immediately (21%, *P* = 0.003) and 1 h after exercise (15.5%, *P* = 0.009) at midtraining and only immediately after exercise (20.3%, *P* = 0.001) at posttraining.

**Figure 7:**
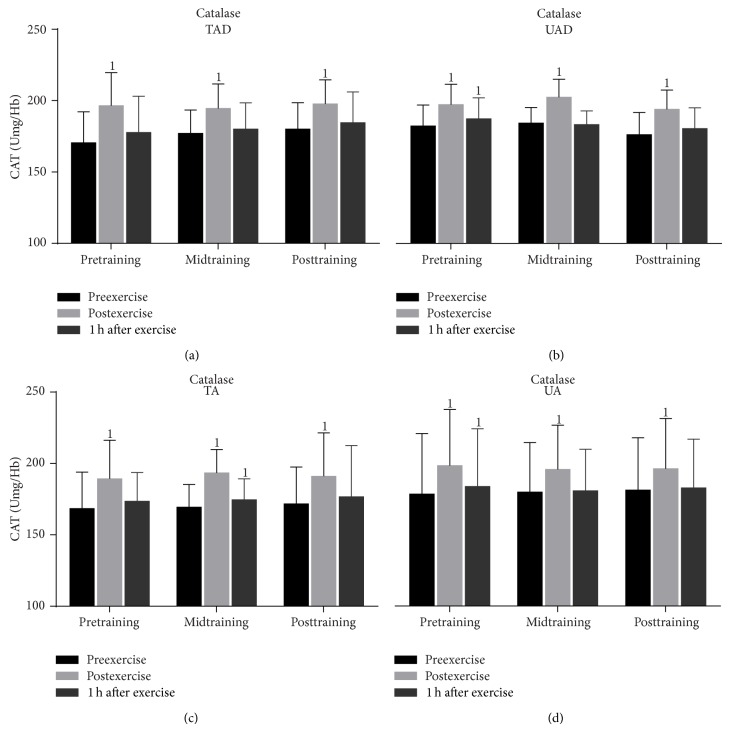
Changes of erythrocyte catalase activity in response to acute exercise and training in adolescents and adult participants. CAT, catalase activity; h, hour; TAD, trained adolescents; TA, trained adults; UAD, untrained adolescents; UA, untrained adults; ^1^significant (*P* < 0.05) difference with baseline values at rest.

Catalase activity (Figure 7) increased significantly (*P* = 0.000) in TAD in response to exercise immediately after exercise and recovered thereafter throughout the experimental period (Figure 7). In TA, exercise induced a rise in catalase activity pretraining (only at postexercise, *P* = 0.000), midtraining (postexercise, *P* = 0.000; 1 h after exercise, *P* = 0.056), and posttraining (only at postexercise, *P* = 0.000). In UAD and UA, exercise increased catalase activity immediately (UAD: pretraining, *P* = 0.000; midtraining, *P* = 0.000; posttraining, *P* = 0.000; UA: pretraining, *P* = 0.000; midtraining, *P* = 0.000; posttraining, *P* = 0.000) and 1 h after exercise (UAD: only at pretraining, *P* = 0.002; UA: only at pretraining, *P* = 0.005) throughout the experimental period.

**Figure 8:**
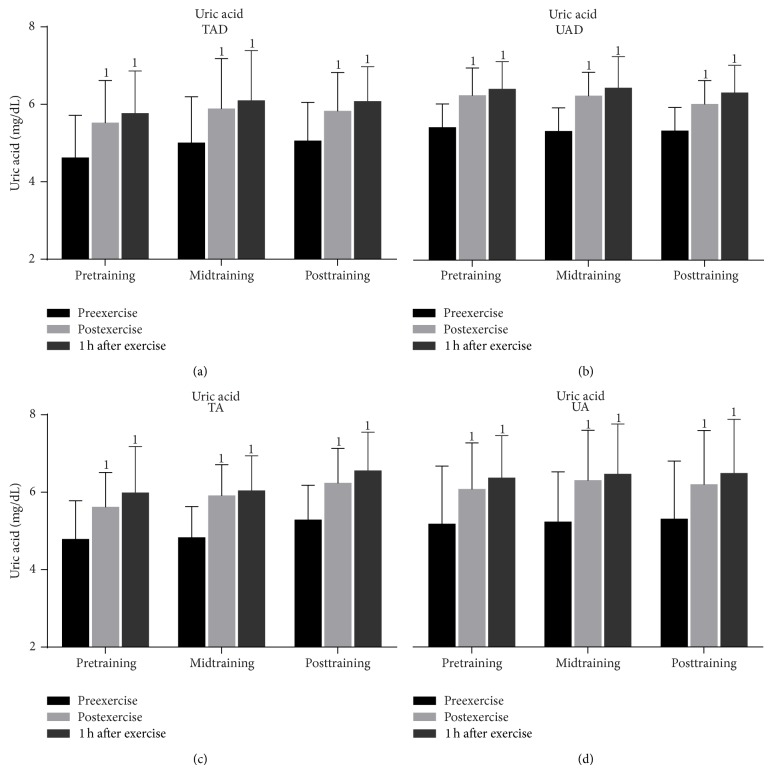
Changes of uric acid concentration in response to acute exercise and training in adolescents and adult participants. h, hour; TAD, trained adolescents; TA, trained adults; UAD, untrained adolescents; UA, untrained adults; ^1^significant (*P* < 0.05) difference with baseline values at rest.

**Figure 9:**
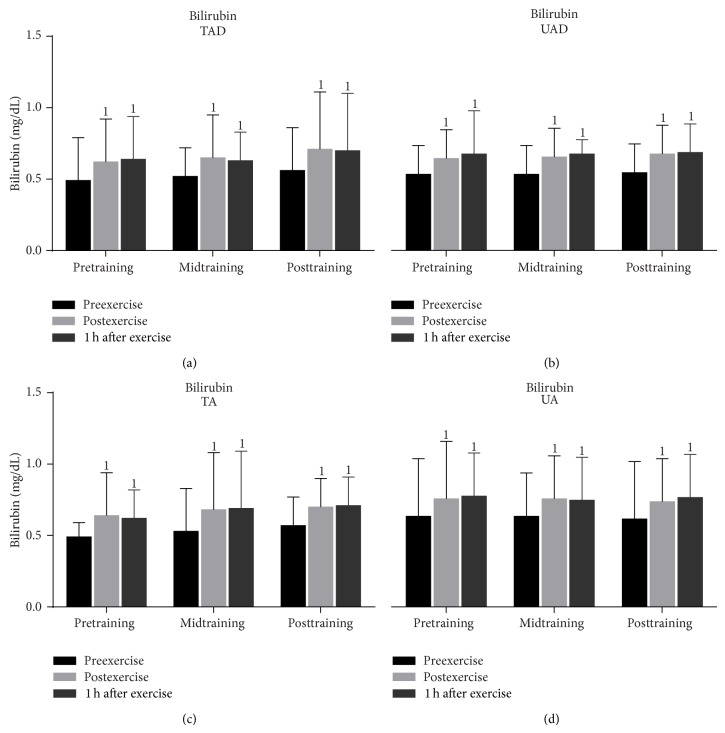
Changes of bilirubin catalase activity in response to acute exercise and training in adolescents and adult participants. h, hour; TAD, trained adolescents; TA, trained adults; UAD, untrained adolescents; UA, untrained adults; ^1^significant (*P* < 0.05) difference with baseline values at rest.

In all groups, exercise induced a significant rise (from 13.0% to 23.0%) of uric acid concentration (Figure 8) immediately after exercise that peaked 1 h after exercise (increase at this time point ranged from 18.0% to 25.0%) throughout the experimental period. Exercise increased (from 19.0% to 30.0%) serum bilirubin concentration (Figure 9) in all groups immediately and 1 h after exercise (increase at this time point ranged from 18.0% to 31.0%) throughout the experimental period.

### 3.2. Age Effects

PC (Figure 3) and TBARS (Figure 4) resting values were comparable between adolescent and adult groups, independent of training status. Exercise-induced changes of PC were similar in TAD and TA whereas UAD demonstrated a lower rise of protein carbonyls than UA immediately and 1 h after exercise throughout the experimental period. TBARS responses to exercise were comparable among adolescent and adult groups.

Resting TAC values (Figure 5) were higher in TA than in TAD at pre- (*P* = 0.009) and midtraining (*P* = 0.009) but not at posttraining. UA demonstrated higher resting TAC values than UAD at pre- (*P* = 0.005), mid- (*P* = 0.05), and posttraining (*P* = 0.032). When the magnitude of exercise-induced change in TAC values was examined, it was revealed that adult participants exhibited a larger TAC rise than their adolescent counterparts immediately and 1 h after exercise throughout the experimental period.

Untrained adults had greater resting GSH values (Figure 6) than UAD throughout the experimental period (34% at pretraining, *P* = 0.034; 27% at midtraining, *P* = 0.032; and 34% at posttraining, *P* = 0.023) whereas TA had higher resting GSH levels than TAD only at posttraining (50%, *P* = 0.001). Exercise-induced GSH decline was more pronounced in TA compared to TAD only at posttraining (immediately after exercise, *P* = 0.001; 1 h after exercise, *P* = 0.001) but there were no differences between UA and UAD at any time.

Resting and exercise-induced changes catalase activity values (Figure 7) as well as uric acid (Figure 8) and bilirubin concentration (Figure 9) were similar in adult and adolescent subjects throughout the experimental period, independent of training status.

### 3.3. Training Responses

When UAD were compared to TAD it was revealed that the former had (a) a greater rise of PC concentration (Figure 3) 1 h after exercise at mid- and posttraining as well as at rest at posttraining, (b) a more pronounced elevation of TBARS (Figure 4) immediately after exercise as well as 1 h after exercise at mid- and posttraining, (c) lower TAC values (Figure 5) at rest as well as in response to exercise at all times throughout the experimental period, and (d) lower GSH values (Figure 6) at rest throughout the experimental period. No differences were detected between these two groups in catalase activity (Figure 7), bilirubin (Figure 8), and uric acid (Figure 9) responses at any time of the experimental period.

When UA were compared to TA it was revealed that the former had (a) a greater rise of PC (Figure 3) in response to exercise at all times throughout, (b) a more pronounced response of TBARS (Figure 4) immediately (*P* = 0.006) and 1 h after exercise (*P* = 0.002) at posttraining, (c) lower TAC values (Figure 5) at rest as well as in response to exercise at all times throughout the experimental period, and (d) lower GSH values (Figure 6) at rest throughout the experimental period as well as immediately (*P* = 0.004) and 1 h after exercise (*P* = 0.001) at posttraining. No differences were detected between these two groups in catalase activity (Figure 7), uric acid (Figure 8), and bilirubin (Figure 9) responses at any time of the experimental period.

## 4. Discussion

The present investigation examined resting and exercise-induced responses in redox status markers in adolescents and adults participating in a year-long running training season. Results indicate that (i) independent of training status both adolescents and adults demonstrate an elevation of oxidative stress and antioxidant status markers in response to acute exercise stress, (ii) independent of training status, adults demonstrated a higher TAC and GSH at rest and a greater change of these two antioxidant status markers after acute exercise, and (iii) untrained adults and adolescents may have a lower antioxidant reserve than their trained counterparts.

A marked improvement of aerobic capacity was noted in all trained participants (independent of age), since VO_2max_, vVO_2max_, and the vAT increased markedly in response to prolonged training. These results are in agreement with previous studies reporting similar changes for both adolescent [35] and adult [36] athletes.

### 4.1. Responses to Acute Exercise

It appears that children and adults responded to acute exercise by increasing considerably their TBARS and protein carbonyl levels for 1 h after exercise independent of training status. This is in accordance with a number of studies that showed an elevation of oxidative stress markers in children, adolescents, and adult humans in response to various exercise protocols and modes [15, 26, 37–40]. Our results and those of others suggest that children and/or adolescent may respond, both qualitatively and quantitatively, to acute submaximal exercise stress similarly to adults [15, 26].

The elevation of oxidative stress markers immediately after exercise may be attributed to the substantial elevation of oxygen processing by the respiratory chain of mitochondria of both adolescent and adult participants in response to our time-trial bout [20]. The time-frame of these responses was similar to that reported for adults using a similar exercise protocol, that is, an elevation of approximately one hour long [26]. The rise of oxidative stress markers 1 h after exercise may be attributed to either an activation of signaling pathways to enhance antioxidant activity or extramitochondrial pathways of ROS production, such as those of myeloperoxidase and elastase (generate superoxide) of circulating leukocytes (i.e., neutrophils), which are recruited and migrated to the injured skeletal muscles [41]. The exercise-induced rise of neutrophils persists for approximately an hour, during which mitochondrial oxygen processing has reached baseline levels again [42]. Although evidence suggests that children may exhibit a different immune response to a physical challenge [43], exercise-induced neutrophilia has been observed in both adults and children [20, 44].

Acute exercise increased TAC and reduced GSH independent of age and training status suggesting a recruitment of antioxidant resources to offset the elevated oxidative stress. Similar findings have been reported by Nikolaidis et al. [15] for prepubescent boys and girls in response to intense swimming protocols as well as in response to other exercise modalities such as basketball [38] and soccer [38]. The erythrocyte GSH decline reveals a potential increased utilization of this antioxidant as a result of exercise-induced RONS. A similar effect has been shown in male and female swimmers [16]. These findings are also in agreement with similar GSH reductions after endurance, dynamic, or interval exercise [4, 26, 44].

Exercise also elicited a marked rise of uric acid in all groups probably due to augmented adenine breakdown during exercise. This finding coincides with the observation of increased oxidative stress, as evidenced by the elevated TBARS and protein carbonyls, also suggesting increased ROS generation [45]. Furthermore, uric acid elevation may have enhanced TAC response to exercise or during recovery [46], since it has been shown to account for approximately 30% of TAC upregulation [47]. In fact, uric acid elevation paralleled that of TAC after exercise in this study.

In agreement with previous reports [40, 48], bilirubin increased after exercise probably due to increased haemolysis on account of repeated foot striking on the ground during running [49]. This bilirubin increase may also have contributed to TAC upregulation [50].

### 4.2. Age Effects

There are only a limited number of studies that compared exercise-induced oxidative stress and antioxidant status responses between adults and adolescents. Previous research has suggested that children and adolescents may release more ROS in response to exercise than adults due to their faster VO_2_ kinetics [18, 19]. However, this was not the case in this study since adults exhibited a greater oxidative stress and antioxidant response to exercise than adolescents. In one of the few studies, Timmons et al. [43] directly compared oxidative stress adaptations in children and young adult males in response to acute cycling exercise of moderate intensity (60–70% of maximal aerobic power). Although, in that study, there were no differences in basal levels of protein carbonyls, a lower response was observed in children than men after exercise due to a blunted cytokine and immune response to exercise in children.

Adults demonstrated also a greater TAC rise and GSH decline than their adolescent counterparts in response to exercise suggesting that they performed a greater exercise overload (volume and intensity). GSH levels may be different between adolescents and adults with higher concentrations being observed in adults [51]. Furthermore, lower CoQ redox ratio (ubiquinol/ubiquinone) was observed in children suggesting an age effect on redox status [52]. This age-dependent upregulation of GSH and redox status warrants further investigation. Nevertheless there are reports in the literature suggesting a more pronounced [53] or insignificant response [14] of antioxidant status markers compared to adults during or after exercise. Variations in age, conditioning status, exercise protocols and modalities, and control of antioxidant nutritional intake among studies may explain these discrepancies.

### 4.3. Training Effects

There is a plethora of evidence suggesting that regular physical activity or training upregulates activity of antioxidant enzymes and DNA repair enzymes in young adults as well as in the elderly at rest or in response to exercise stress [21, 23, 54]. Although there were no differences in basal oxidative stress levels, as evidenced by TBARS and protein carbonyl levels, between trained and untrained participants, the former demonstrated a greater TAC and GSH levels at rest and in response to acute exercise. GSH is the most important thiol in the human body and acts as a substrate for glutathione peroxidase in peroxidase ROS inhibition [55] and can directly detoxify ROS and enhances the functional antioxidant capacity of vitamins C and E [56]. Indeed, systematic exercise training elevates GSH concentration and the activity of GSH-dependent enzymes [55]. In an elegant work, Kihlstrom [57] showed that the enhanced capacity of the rat myocardium to detoxify ROS following swimming training is mainly attributed to the training-induced elevation of GSH, reduction of GSSG reductase activity, and rise of the activity of thioredoxin reductase. Similar findings were obtained by animal training studies that used running as the exercise mode [58]. However, evidence of training-induced effects on oxidative stress and antioxidant status markers in adolescents is scarce.

A major finding of this study is the greater TAC and GSH reserves in trained adolescents as compared to their sedentary counterparts. This TAC and GSH response to training was similar to that observed in adult populations. This training effect was more pronounced after the second part of training (midtraining), a finding that may be explained by the greater training overload adapted during that period which plays a crucial role in the enhancement of the antioxidant defense system [59]. Similar TAC increases have been seen in response to a rugby season characterized by periodized fluctuations in training load [60]. Since uric acid represents at least one-third of exercise-induced rise in TAC [61] one would expect to see a training-induced increase of uric acid. Even though others observed a training-induced rise of uric acid in blood and in muscle of adult subjects [60], such adaptation was not seen in this study. Possibly, other nonenzymatic water soluble antioxidants that were not assessed in this study (i.e., ascorbic acid) may have contributed to the increase in TAC due to training.

Similar GSH adaptations have been obtained in response to low (17–43 km·week^−1^) and high (80–147 km·week^−1^) training running volume [62]. Kabasakalis et al. [25] failed to observe a similar effect on TAC in response to prolonged swimming training. However, no details of the training regime (i.e., intensity, training load, etc.) that was followed throughout the year were given in that study. Furthermore, no significant differences have been reported between elite adolescent swimmers and moderately trained ones [63]. The discrepancies between those two studies and the present investigation may be related to the conditioning status of participants (elite runners versus moderately trained) and exercise training mode (running versus swimming). In contrast, Gougoura et al. [64] showed that trained children had lower GSH levels and antioxidant capacity in blood than untrained ones. However, direct comparison of that study may not be feasible due to differences in participants' age.

Catalase activity remained unaltered by training in this study. Similar findings were reported by others after four and 13–23 weeks of training [25, 65]. In contrast to these studies, lower catalase levels were found in trained adolescents when compared to untrained age-matched controls [66]. On the other hand, cross-sectional studies revealed a greater catalase activity in adult trained distance runners [62] and cyclists [67] compared to untrained individuals. Similar findings have been obtained by training studies [68]. Differences in type of training modality, age, training level, and duration of study, as well as nutritional status of the participants, can largely account for these discrepancies. Further investigation is needed in order to elucidate how training affects antioxidant enzymes in the adolescent population.

Trained adolescents demonstrated similar basal levels and greater exercise-induced responses of TBARS and protein carbonyls. In contrast to our results, cross-sectional studies have reported both lower [69] and higher basal lipid peroxidation levels for trained children, adolescents, and adults compared to untrained ones [63, 64]. Cavas and Tarhan [65] reported higher lipid peroxidation following a 4-week swimming training regardless of an increase in several antioxidant parameters (superoxide dismutase, catalase, and glutathione). On the other hand, lower protein carbonyls have been reported following training in adults [27]. Differences in research designs as well as in the time of sampling may explain these discrepancies. The greater level of oxidative stress markers in trained participants following exercise may be attributed to the greater exercise load obtained during exercise testing.

## 5. Conclusions

In summary, aerobic training appears to cause an improvement of the antioxidant system in adolescents in a fashion similar to that seen in adults. Likewise, age may also be a determinant factor of oxidative stress and antioxidant status adaptations at rest and in response to exercise. Proper training program development and implementation could lead to an upregulation of some components of the antioxidant defense system or reserve and further studies are needed to delineate the effects of chronic training on redox status of adolescents.

## Figures and Tables

**Table 1:** Participants' anthropometric, physiological, and training characteristics (mean ± SD).

	Adolescents		Adults	
	Trained (*n* = 13)	Control (*n* = 11)	Trained (*n* = 12)	Control (*n* = 10)
Age (yrs)				
Baseline	14.1 ± 1.1	14.8 ± 0.9	25.2 ± 6.8^3^	27 ± 6.0^4^
Body mass (kg)				
Baseline	54.1 ± 8.5	62.4 ± 8.4^1^	66.9 ± 6.0^3^	71.4 ± 12.4^2,4^
Midseason	55.1 ± 7.9	63.3 ± 6.9^1^	65.4 ± 5.8^3^	71.8 ± 9.7^2,4^
End-season	53.7 ± 7.5	63.9 ± 7.3^1^	64.7 ± 8.4^3^	72.3 ± 10.1^2,4^
Height (cm)				
Baseline	165.6 ± 7.4	171.1 ± 9.4^1^	175.1 ± 4.4^3^	174.2 ± 9.0^4^
Midseason	167.8 ± 7.1	172.2 ± 8.5^1^	175.2 ± 3.9^3^	174.4 ± 9.3^4^
End-season	168.1 ± 7.6	173.5 ± 11.2^1^	175.3 ± 4.2^3^	174.4 ± 8.1^4^
Body mass index				
Baseline	19.4 ± 2.1	21.2 ± 1.4^1^	21.8 ± 1.7^3^	23.1 ± 3.3^2,4^
Midseason	19.5 ± 2.3	21.3 ± 1.8^1^	21.3 ± 1.8^3^	23.6 ± 2.9^2,4^
End-season	19.0 ± 2.1	21.2 ± 1.5^1^	21.1 ± 1.8^3^	23.7 ± 2.8^2,4^
Body fat (%)				
Baseline	6.2 ± 2.8	12.6 ± 2.7^1^	8.2 ± 3.8^3^	11.8 ± 4.8^2,4^
Midseason	5.6 ± 2.1	12.9 ± 3.3^1^	7.9 ± 4.6^3^	12.1 ± 5.2^2,4^
End-season	5.7 ± 2.5	12.8 ± 3.5^1^	7.8 ± 4.8^3^	11.9 ± 4.9^2,4^
Tanner stages				
Baseline	2-3	2-3	N/A	N/A
Midseason	2-3	2-3	N/A	N/A
End-season	2-3	2-3	N/A	N/A

A significant difference with baseline (*P* < 0.05); a significant difference between midseason and end-season (*P* < 0.05); ^1^a significant difference between adolescent groups at the corresponding time point (*P* < 0.05); ^2^a significant difference between adult groups at the corresponding time point (*P* < 0.05); ^3^a significant difference between trained groups at the corresponding time point (*P* < 0.05); ^4^a statistical difference between control groups at the corresponding time point (*P* < 0.05).

**Table 2:** Mean daily energy intake (mean ± SD).

	Adolescents		Adults	
	Trained (*n* = 13)	Control (*n* = 11)	Trained (*n* = 12)	Control (*n* = 10)
Energy (Kcal)				
Baseline	2,269.8 ± 259.7	2,065.1 ± 213.6	2,896.7 ± 351.2	2,607.4 ± 309.8
Midseason	2,312.5 ± 271.3	2,091.0 ± 238.4	2,836.7 ± 346.9	2,626.3 ± 292.7
End-season	2,334.4 ± 265.9	2107.2 ± 243.3	2,827.5 ± 319.3	2,355.4 ± 326.1
Carbohydrates (% energy)				
Baseline	62.4 ± 4.6	61.6 ± 5.9	61.8 ± 6.4	58.9 ± 9.7
Midseason	61.1 ± 6.1	62.3 ± 7.5	62.5 ± 7.9	59.8 ± 10.4
End-season	61.7 ± 5.2	60.6 ± 8.3	60.9 ± 8.1	59.1 ± 8.8
Fat (% energy)				
Baseline	22.4 ± 3.8	24.0 ± 4.5	23.4 ± 3.5	26.7 ± 6.2
Midseason	23.1 ± 3.2	22.9 ± 3.6	21.8 ± 3.0	25.4 ± 5.5
End-season	22.2 ± 4.3	25.2 ± 5.1	23.8 ± 4.2	26.2 ± 5.1
Protein (% energy)				
Baseline	15.2 ± 1.6	14.4 ± 1.8	14.8 ± 2.8	14.4 ± 2.6
Midseason	15.8 ± 2.1	14.8 ± 1.7	15.7 ± 2.3	14.8 ± 1.7
End-season	16.1 ± 2.4	14.2 ± 1.5	15.3 ± 1.9	14.7 ± 1.4
Vitamin A (ug, RE)^*^				
Baseline	724.1 ± 196.7	755.2 ± 214.8	794.1 ± 246.3	785.2 ± 263.6
Midseason	752.8 ± 182.9	773.0 ± 230.4	805.2 ± 276.3	793.7 ± 253.1
End-season	744.5 ± 213.0	771.2 ± 209.1	801.1 ± 283.4	810.6 ± 25.9
Vitamin C (mg)				
Baseline	92.5 ± 15.1	90.8 ± 19.6	109.7 ± 23.7	108.3 ± 21.3
Midseason	93.1 ± 18.2	88.4 ± 19.4	111.4 ± 28.1	106.8 ± 24.9
End-season	90.6 ± 17.6	91.9 ± 18.5	108.2 ± 30.6	100.5 ± 26.8
Vitamin E (mg, a-TE^†^)				
Baseline	6.9 ± 2.7	7.3 ± 2.2	7.1 ± 3.0	7.5 ± 2.6
Midseason	7.2 ± 3.6	7.0 ± 4.1	7.4 ± 3.8	7.7 ± 3.3
End-season	6.8 ± 3.4	7.1 ± 3.9	7.3 ± 3.5	7.6 ± 4.1
Selenium (ug)				
Baseline	75.8 ± 16.2	77.9 ± 14.6	81.3 ± 20.5	83.4 ± 21.6
Midseason	79.3 ± 24.8	75.6 ± 22.5	84.2 ± 23.3	80.9 ± 20.8
End-season	76.7 ± 22.1	81.2 ± 33.7	82.7 ± 28.0	84.4 ± 26.3

^∗^RE: retinol equivalents; ^†^a-TE: alpha-tocopherol equivalents.
